# Intelligent Evaluation System of Government Emergency Management Based on BP Neural Network

**DOI:** 10.1109/ACCESS.2020.3032462

**Published:** 2020-10-20

**Authors:** Dian Jia, Zhaoyang Wu

**Affiliations:** 1 School of Economics and ManagementQinghai Nationalities University 290266 Xining 810007 China; 2 School of Economics and ManagementQinghai Normal University 107627 Xining 810016 China

**Keywords:** BP neural network, government emergency, emergency management, intelligent evaluation system

## Abstract

With the deepening of the global economic community, various emergencies emerge in endlessly, and the risks gradually increase. People’s lives and property are threatened, which also causes a great burden on the social economy. Hitherto unknown novel coronavirus events occurred in China after the outbreak of the new coronavirus in 2019. The emergency management system is not perfect, so we start to study and improve the deficiencies of the emergency management system, but it is still difficult to effectively prevent and deal with all kinds of sudden and frequent social problems. Therefore, this paper puts forward the research of intelligent evaluation system of government emergency management based on BP neural network. In this paper, an intelligent evaluation system of government emergency management based on Internet of things environment is established, and then the system is deepened by BP neural network algorithm to avoid the interference of human factors. An objective intelligent evaluation system of government emergency management is constructed and verified by an example. We applied the system in a province, and proved that the system has strong executive ability, outstanding big data computing ability, and can objectively evaluate and analyze the government emergency management. The operability and accuracy of the intelligent evaluation system are verified. The effective evaluation content provides a new idea and method for government emergency management. And then continuously improve the emergency management measures to achieve the effect of dealing with things smoothly without panic.

## I. Introduction

The 19th National Congress of the Communist Party of China [1] pointed out that protecting the safety of Chinese people’s lives and property and improving people’s sense of happiness and income are the top priorities of the government’s work. At present, China is in a period of social transformation. In the process of structural adjustment and poverty alleviation, social contradictions have also been highlighted. All kinds of emergencies occur frequently, which poses a serious threat to the safety of people’s lives and property. “Since the beginning of the 21st century, the incidence of mass emergencies in China is relatively high.” A series of novel coronavirus occurred successively: after the 1998 flood, the “3.1” violent terrorist cases in 2013 and the “5.22” terrorist attacks in Urumqi in 2014, the wreck of the “Oriental Star” in 2015, the prisoners escaped in 2017, the African swine fever in 2018, and the new coronavirus pneumonia in the Songhua River. The emergence of emergencies reminds the government to do a good job in the accumulation of emergency knowledge, improve the ability of the government to respond to emergencies, ensure the safety of people’s lives and property, and lay the foundation for building a strong socialist country [2].

Due to the danger, uncontrollability and diversity of emergencies, experience and enthusiasm alone cannot meet the needs of local governments to deal with emergencies [3]. After years of research and practice, the education and management theory in western countries has made great progress, while the emergency education and management theory in China is relatively weak. Although many scholars have recognized the importance and necessity of emergency management for social development, many scholars in this field have also put forward the framework of emergency management, but for the continuous development of emergency management, China has actually made great progress, but it is still insufficient. At present, the emergency management measures in operation need to be discussed, which cannot meet the actual needs in theory. At present, China is in the stage of discipline diversification, and is studying how to construct the management system and mechanism of crisis response. In 2005, the State Council promulgated the emergency plan for public emergencies [4], which stipulates that “social organizations, enterprises and institutions, volunteers and other social forces should be actively mobilized to participate in the emergency rescue work.” In addition, the Third Plenary Session of the 18th Central Committee of the Communist Party of China also proposed to “improve the way of social governance, stimulate the vitality of social organizations, innovate the system of effective prevention and resolution of social contradictions, and improve the public security system”. On the basis of crisis management in developed countries, further strengthening the research on emergency management of Dalian municipal government can broaden the vision of Fujian municipal government and enrich the theory of emergency management of municipal government.

According to the diversity and complexity of emergency research objects, scholars in different fields have analyzed and studied emergencies from three aspects of individuals, organizations and governments. From the perspective of emergencies in recent years, although there are differences in causes and degrees, there is a common point, that is, uncontrollable [5]. The destructiveness and urgency after the outbreak of the epidemic is easy to become the focus of public opinion; if it is not handled properly, it is more likely to cause more secondary damage, the image of the government is damaged, and the legitimacy is questioned by the public. The effect of emergency handling is not only the evaluation of the emergency management ability of the municipal government, but also the test of its daily management ability. Rome was not built in a day. How to deal with emergencies is a systematic work, not a simple post event response. We should do emergency management before, during and after the event.

How to establish the evaluation index system of emergency management intelligence, the actual situation of emergency management in the underdeveloped areas of Northwest China, learn from the beneficial achievements at home and abroad, use the facts and data to find out the shortage of government emergency management ability, and put forward the corresponding construction path [6]. In other words, it is an important subject to build the government’s emergency management ability in an all-round way. Based on the current situation of our country, combined with the research of many scholars, this paper analyzes the current situation of government emergency management. Strengthening the research on the construction of emergency management capacity is not only beneficial to the emergency management system of the county-level government, but also helpful to create a close image of the government and the people and enhance the public’s trust in the government [7].

Throughout ancient and modern times, the research on emergency management is a blank in ancient China. The real starting point may be the 1970s, but the research at that time was still superficial and lacked depth, especially in theory and practice. Until the 1990s, some experts and scholars began to conduct in-depth and systematic research on emergency crisis management and achieved certain results. For example, related researchers published “Research on the coping methods of group emergencies and social crisis events” [6], and “emergency management of emergencies” co-authored by many scholars [7]. These works are specific in the level of emergency crisis management related theoretical knowledge, which has a good reference significance for further improving the theoretical experience.

The innovation of this paper is as follows: first, according to the needs of the times. Under the special background of symbiosis with epidemic situation, it is more urgent to improve the emergency management system of each province. And in today’s Internet of things, this study can be based on the Internet of things government emergency management intelligent evaluation system, for local emergencies to give corresponding measures; second, the research perspective is novel. In this paper, the comprehensive use of literature analysis, case confirmation, questionnaire and other forms of subject demonstration, can test the feasibility of the system; the system is applied to a province, and then through field investigation to understand the emergency management level of each region. Comprehensive analysis and evaluation of the regional emergency management mechanism deficiencies provide useful countermeasures to improve the ability of local government emergency management.

## II. Overview of Research Contents, Methods and Related Concepts

This paper takes a province as an example, applies the established emergency system to the province, and compares the evaluation authenticity before and after deepening without using BP neural network algorithm. Referring to the previous research content, taking the reflection of government and other official departments as the relevant standards of China’s emergency system, a series of emergency response systems are established and sublimated.

### A. Research Content

(1) Research on intelligent evaluation method of emergency management: Based on the research on the evaluation index system and evaluation method of emergency management at home and abroad, an intelligent evaluation method of emergency management based on BP neural network is proposed [10]. Through the comparison of several algorithms, we decided to use BP neural network algorithm. According to the intelligent evaluation index system of emergency management, the BP neural network model is designed the feasible evaluation program is given. In the calculation method, the BP neural network toolbox is used to design and calculate the network based on the Internet of things [11]. Through training and testing the learning samples, the error of the model can reach the predetermined range. Finally, an example is given to verify the effectiveness and operability of the method.
(2) Design of intelligent evaluation system for emergency management: if we want to evaluate the government’s response more accurately, we must understand the government’s emergency management system architecture. Selecting indicators with scientific basis, the index with complete structure helps to meet the integrity, effectiveness and feasibility of the system, and at the same time, it is relatively easy to build. In terms of time, we start from the beginning, trim, add, use to upgrade again. From the aspect of method use, we should test the effectiveness of different algorithms in this system resume for many times, and then pay attention to maintaining various standards. From the idea, we need to step by step, pass the experimental test many times, and get careful thinking.
(3) The establishment of emergency management intelligent evaluation system: taking a province as an example, the original data of all regions in the province are taken as the experimental objects. The development index of Internet of things is used to evaluate the emergency management intelligence of each region [12].

### B. Internet of Things Technology

Internet of things technology [13] has triggered the third wave of information industry. Its original idea is to realize the interconnection between objects. Combining physical entity with virtual technology, using intelligent sensor and other identification sensing technology, real-time collection of entity information and transmission to the Internet, all kinds of information of objects are linked together [14]. Through the computer processing demand and resource supply and demand relationship, optimize resource allocation, realize the intelligent identification and management of all projects. With the development of the Internet of things, the Internet of things from the simple network of goods to a broader connotation. The Internet of things realizes the interconnection of any object at any time and any place. It can be seen as the deep integration of digital world, virtual network world and real physical world, and it is the direction of social science and technology development in the future.

### C. BP Neural Network

The self-learning function of BP artificial neural network [15] is used to fit the complex curve, which can be used for the evaluation of government emergency management. The method is simple and accurate, avoids the influence of personal subjective characteristics, and the evaluation results have strong objectivity and comprehensiveness. At the same time, due to the complexity of the government emergency management evaluation index system, there may be a hidden correlation between multiple indicators. The premise of other evaluation methods is that the indexes of the established index system are not related, while BP artificial neural network has better fault tolerance. Through continuous training, we can avoid the inaccurate evaluation results caused by the internal correlation between indicators and make the evaluation more scientific and reasonable.

BP neural network is a computational structure characterized by simulated biological process and showing local characteristics of human brain [16]. $U_{i} $ is the linear combination of input signals, and output $v_{i} $ is the local sensing area of neurons, $X_{i} $ is the input signal of neurons, and $W_{ij} $ is the synaptic strength.

\begin{equation*} U_{i} =\sum \nolimits _{j} {W_{ij} X_{i}}\tag{1}\end{equation*}

Output $Y_{i} $ of neurons $F()$:

\begin{equation*} y_{i} =F\left({\sum \nolimits _{j} {W_{ij} X_{i}} +b_{i} }\right)\tag{2}\end{equation*}

$F()$ can select different functions according to different function types, and its typical functions are as follows.

(1) Threshold function [17]: the threshold function is called jump function, and the output value of neurons is 1 or 0, which reflects the excitation or inhibition of neurons.
  \begin{align*} F(v)=&\begin{cases} {1,}&{v\ge 0} \\ {0,}&{v< 0} \\ \end{cases} \tag{3}\\ F(v)=&\begin{cases} {1,}&{v_{i} \ge 0} \\ {-1,}&{v_{i} < 0} \\ \end{cases}\tag{4}\end{align*}
(2) Piecewise linear function [18]: the function belongs to the nonlinear function in the linear region, [−1,1].
  \begin{align*} F(v)=\begin{cases} {1,}&{v_{i} \ge 1} \\ {v,}&{1>v>-1} \\ {-1,}&{v_{i} \le -1} \\ \end{cases}\tag{5}\end{align*}
(3) Sigmoid function [19]: very different from other functions. In fact, this function is one of the commonly used excitation functions, also known as $S$ type function.
  \begin{equation*} F(v)=\frac {1}{1+\exp (-av)}\tag{6}\end{equation*}

In the network, the mathematical relationship between the various parts plays a great role. First, the front-end part, also known as the input layer, acts on the front-end part through $O_{k} $ and $net_{k} $.

\begin{align*} O_{k}=&F(net_{k}),\quad k=1,2,\ldots,n \tag{7}\\ net_{k}=&\sum \nolimits _{j=0}^{m} {w_{ij}} y_{i},\quad k=1,2,\ldots,n\tag{8}\end{align*}

Secondly, the middle part, also known as hidden layer, influences the middle part through $y_{i} $ and $net_{k} $.

\begin{align*} y_{i}=&F(net_{k}),\quad j=1,2,\ldots,m \tag{9}\\ net_{k}=&\sum \nolimits _{i=0}^{m} {jv_{i}},\quad j=1,2,\ldots,m\tag{10}\end{align*}

Unipolar $S$ type function is a kind of function. At the same time, the transfer function $F(x)$ is applied to the neural network. Unipolar $S$ type functions can be selected by $F(x)$.

\begin{equation*} F(x)=\frac {1}{1+e^{-x}}\tag{11}\end{equation*}

Continuous function has continuity and differentiability, which makes the application of function more extensive. $F(x)$ has this property.

\begin{equation*} F(x)'=F(x)[1-F(x)]\tag{12}\end{equation*}

The other is bipolar $S$ function, which has its own characteristics and is widely used [20].

\begin{equation*} F(x)=\frac {1-e^{-x}}{1+e^{-x}}\tag{13}\end{equation*}

Finally, the three-layer BP neural network is composed of the above parts.

### D. Overview of Related Concepts

(1) Emergency [21]: as the name implies, it refers to some unexpected or unforeseen events, such as sudden natural disasters or man-made disasters. Emergency response mainly includes natural disasters, public health accidents, social security affairs and accident disasters [22]. The emergency treatment of these emergencies generally goes through five stages: brewing, outbreak, diffusion, recovery and sequelae. “The sooner we deal with the crisis, the better. There is no need to wait for the crisis to reach its breaking point. Therefore, the core of crisis management is to grasp the opportunity to deal with the crisis.” Now it has entered the era of big data [23]. With the rapid development of the Internet of things, once the public opinion guidance measures are not timely and accurate, it may lead to new and more serious events. Therefore, it is often important to make effective use of the media to convey the latest information and events to the public in a timely manner, such as holding a press conference, so as to ensure the correct direction of public opinion. Take the right way to deal with emergencies, as far as possible to reduce the possible harm after the incident, so as to achieve the minimum.
(2) Emergency management: emergency management mechanism refers to the structure and operation mechanism of management system. The management system here includes a series of management activities, such as management objects, processes, internal relations, etc. In essence, the management mechanism is the internal relationship, function and operation principle of the management system, and is the core issue to determine the management effect. At the same time, the management mechanism is based on the objective laws and organizational structure and consists of several sub mechanisms.

### E. Technical Route

**FIGURE 1.**
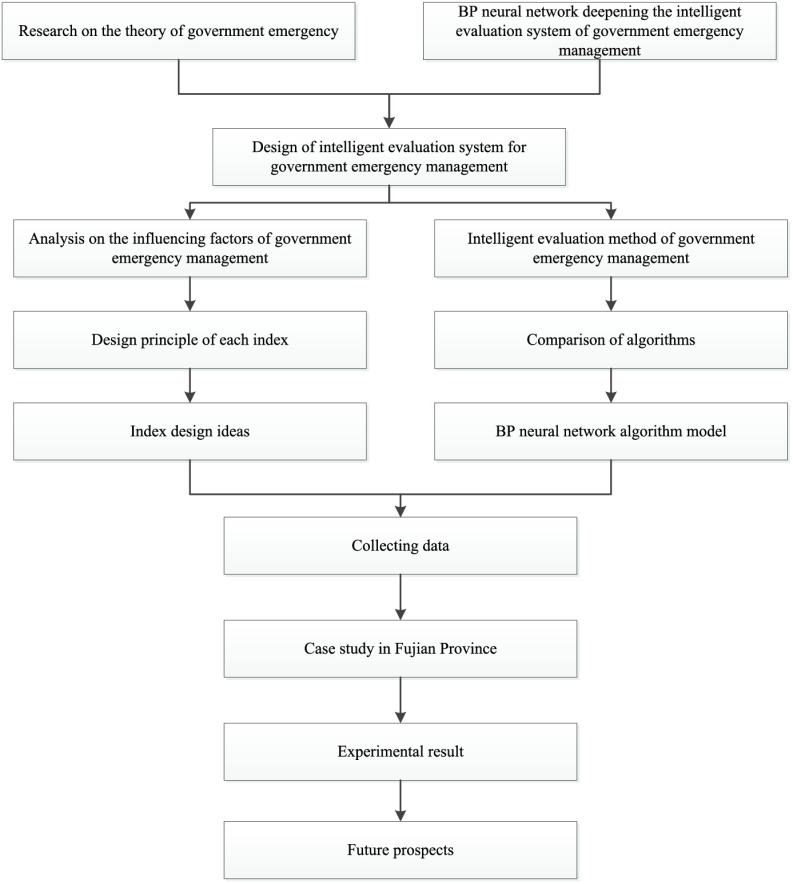
Architecture of this paper.

Shown as Figure 1, this paper deepens the intelligent evaluation system of government emergency management by studying the relevant theories of government emergencies and BP neural network. First of all, through the analysis of the influencing factors of government emergency management; then lists out the government emergency management intelligent evaluation methods that can be used, carries on the comparative analysis, obtains the BP neural network algorithm has the promotion function to the system; finally based on the Internet of things corresponding to the emergency management intelligent evaluation system [24], in line with the standards to improve the emergency management intelligence The reliability of the system and the feasibility of its evaluation content are verified by an example.

## III. Operation Process

### A. Information Collection Link of Internet of Things

The Internet of things is to exchange and communicate information between the network of a commodity and the Internet [25], and realize intelligent identification, positioning, tracking, monitoring and management through various information sensing devices such as radio frequency identification, infrared sensor, global positioning system, laser scanner according to the protocol. The system is based on the Internet of things RFID identification [26] and passive sensing technology [27], providing a unique identity tag for each emergency. Passive sensing technology processes the detected information and sends it through wireless data transmission module. When there is an urgent need, the system can intelligently sense whether the equipment performance of local natural disasters is in good condition, and send voice prompt information, improve the standardized equipment rack of emergency management personnel, ensure safety, and realize the effective connection between natural disasters and equipment [28]. In terms of daily management, the system can collect real-time information about the types of emergency equipment library, the equipment that can master the utilization rate, maintenance rate and damage rate, and provide data to support the emergency management department to optimize and adjust the equipment reserve plan, so as to comprehensively improve the equipment management level of intelligence. The test results of a province fully prove the application value of Internet of things technology in the intelligent evaluation system of emergency management and provide experience for promoting the intelligent evaluation system of emergency management based on Internet of things in China.

### B. BP Neural Network Algorithm

BP neural network algorithm can be combined with these two methods to enter the grey multi-level analysis method. It is used to evaluate the emergency management system, ensure the effectiveness and scientificity of the evaluation, and it has a strong adaptability evaluation of emergency management measures. When the algorithm is used to calculate the weight [29]:

\begin{equation*} AW=\lambda _{\max } W\tag{14}\end{equation*}

In formula (1), $\lambda _{\max } $ represents the largest eigenvalue in $A$ and $W$ represents the direction vector of $\lambda _{\max } $.

Consistency test $CI$:

\begin{equation*} CI=\frac {\lambda _{\max } -m}{m-1}\tag{15}\end{equation*}

Judge the consistency ratio of test data $CR$:

\begin{equation*} CR=\frac {CI}{RI}\tag{16}\end{equation*}

If $CR< 0.01$ is calculated [30], the test result is satisfactory.

In this paper, BP artificial neural network is used to establish the intelligent evaluation model of emergency management, which can not only get rid of the influence of human factors and fuzzy randomness, but also ensure the accuracy of the evaluation. In order to correctly analyze the risk level of emergency management system and effectively implement the risk management in the operation process of emergency management system, an effective evaluation system is established

### C. Overall Design of Intelligent Evaluation System for Emergency Management

Through the case analysis of a province, we have a general understanding of the current situation of emergency management, but to objectively and accurately evaluate the emergency management ability of a provincial government, we must establish a reasonable and comprehensive index system of emergency response capacity. This chapter will describe the evaluation elements, the basis for the construction of the index system, the content of the index system, build an objective, comprehensive and reasonable index system of emergency capability, and explain the selection of the method to determine the weight of each level index.

The determination and selection of evaluation elements is the basis of constructing the evaluation model, which is of great significance to the evaluation model and has an important impact on the evaluation and construction process of the emergency management capacity of government in the later stage. Therefore, before constructing the index system and evaluation model, it is necessary to define the evaluation elements. Evaluation elements can be divided into: subject (Comprehensive Evaluation), object (in the evaluation model, the object corresponding to the evaluation subject is also the evaluation object selected for the problem to be solved in this paper), standard (evaluation basis of the object) and method (efficient evaluation method).

By summarizing a large number of emergency management literature, a complete set of intelligent evaluation index system of government emergency management is established, including 3 first-class indicators and 15 second-class indicators.

The indicators are as follows:

(1) Emergency preparedness included: planning specification, facilities construction, emergency support, system construction and emergency education;
(2) The emergency warning force included: hidden danger investigation force, early warning release force, early warning response force, on-site investigation force and disaster recovery force;
(3) Emergency response capabilities: unified command, information disclosure, social participation, on-site control, emergency rescue

### D. BP Neural Network Deepening Emergency Management Intelligent Evaluation System

In the environment of Internet of things technology, based on the information collection of sensors, BP neural network is used to carry out forward calculation and reverse error analysis. Because BP neural network hidden in the two-layer network, the network node is composed of two layers of array to become AAA. Neural network, also known as multilayer feedforward network, is trained and trained according to error back-propagation algorithm. It can train and co-exist a large number of input-output mode mapping relations without knowing the mathematical equation representing the mapping relationship in advance. The standard neural network uses steepest descent method for learning and training. According to the back-propagation error, the weights and thresholds of the network are gradually adjusted to minimize the sum of squares of the whole neural network errors. Finally, a neural network composed of input layer, hidden layer and output layer is formed. The algorithm includes two processes: forward propagation of data stream and backward propagation of error. By setting the structure of the neural network and the weight and threshold of the last iteration, the output of each neuron is calculated, and the input layer, hidden layer and output layer of the propagation order of the neural network are calculated. The results of each layer of neurons only affect the neurons of the next layer. In the back propagation of error, the influence of each weight and threshold on the total error is calculated from the last layer of neural network, and then corrected. The two processes continue to search for a set of weight vectors in the weight vector space to minimize the network error function, and finally complete the process of information extraction and storage.

Generally speaking, the training process of BP neural network model is actually the error function. In the process of parameter connection weight and threshold optimization, we hope to find the optimal connection weight and threshold minimization error function for the whole training data set. In BP neural network algorithm, the gradient descent method is used to search the negative gradient direction of the parameter error function of the optimal solution. If the gradient of the error function at a certain point is 0, then the error function reaches the minimum value at that point. But in fact, we don’t know whether the minimum is a global minimum or a local minimum. If the error function itself is not only a global minimum, but also has multiple local minima, then the gradient descent method is easy to make the search fall into a local minimum and not find the global minimum. In this case, we need another method to jump out of the local minimum, such as simulated annealing or stochastic gradient descent method, which can help the model approach the global minimum better.

Finally, BP neural network algorithm is applied to the intelligent evaluation system of emergency management, but because the evaluation content of the system is a comprehensive evaluation, there is only one neuron at the output end. Therefore, firstly, the process of index establishment is carried out, the simulated sample data are collected, and the BP artificial neural network is established to determine the network parameters and network structure. At the beginning of network training, according to the learning process in the diagram, the BP artificial neural network of the intelligent evaluation model of emergency management is lower than the setting error through continuous self-learning neural network. At this time, the trained BP neural network is applied to the intelligent evaluation of emergency management, and the emergency management intelligent evaluation model based on BP neural network is established.

## IV. Results and Discussion

### A. Evaluation Algorithm Comparison Results

**FIGURE 2.**
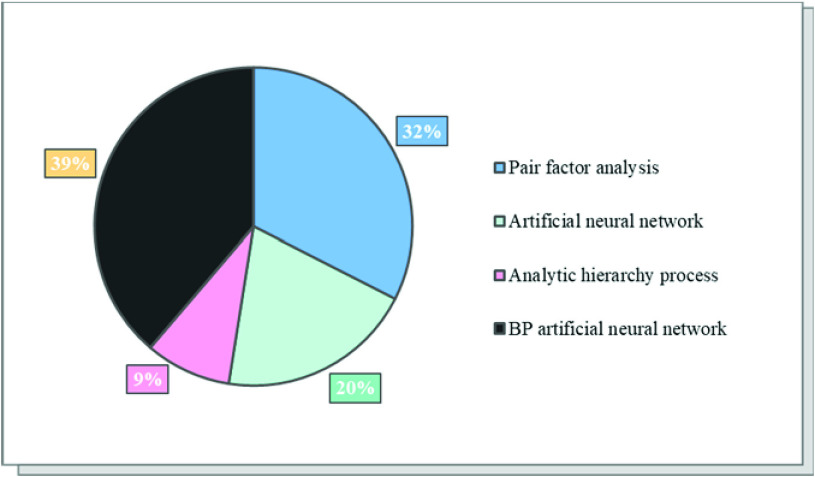
Comparison of applicability scores of four different algorithms.

Shown as Figure 2, we analyze the advantages and disadvantages of the four algorithms. Finally, by consulting many references, we conclude that BP neural network algorithm has the strongest applicability in this system, accounting for 39% of the whole system. At the same time, it can be replaced by factor analysis, accounting for 32% of the total. Although the artificial neural network accounts for 20%, its principle is very similar to that of BP neural network, but due to uncertain factors, we have relatively low scores on its applicability. BP neural network method can quickly adjust the parameters to be corrected, so as to adapt quickly with the change of data.

### B. Score of Intelligent Management System of a Provincial Government

Shown as Table 1, the intelligent evaluation score of the system is 0–10.

**TABLE 1:** Scoring Criteria of Intelligent Evaluation System for Emergency Management Capability of a Provincial Government

Content	Optimal	Good	Commonly	Bad
Score	[8,10]	[6,8)	[5,6)	[0,5)

**FIGURE 3.**
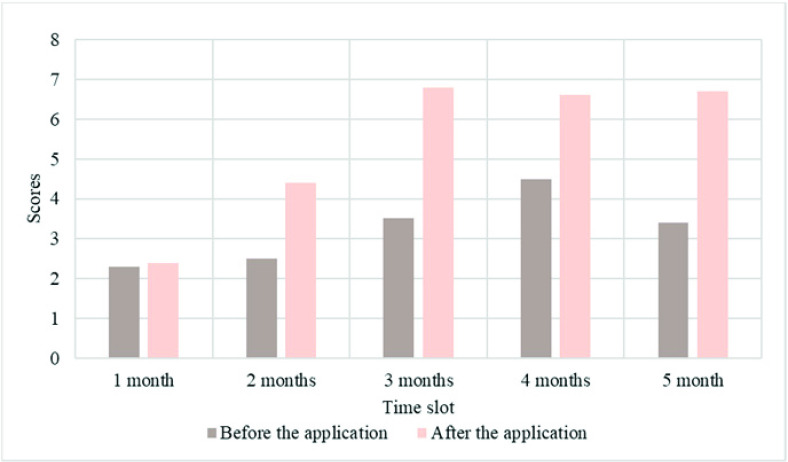
Score results of government emergency management intelligent evaluation system in a province over time.

Shown as Figure 3, we applied the emergency management evaluation system deepened by BP neural network algorithm to various regions in a province and compared the scores of staff questionnaire survey before and after the application of the system. We conducted a six-month test and made statistics on the score of each month. It is known that the system has been continuously improved, and the score of the staff is relatively high, until the third month, the score is the highest. After several months, the score is relatively stable, compared with before the application of the system, the overall score is higher, and the score shows an upward trend. It shows that the system needs to be upgraded continuously during the testing process.

### C. Training Samples of Intelligent Evaluation System for Emergency Management of Local Governments in a Province

**FIGURE 4.**
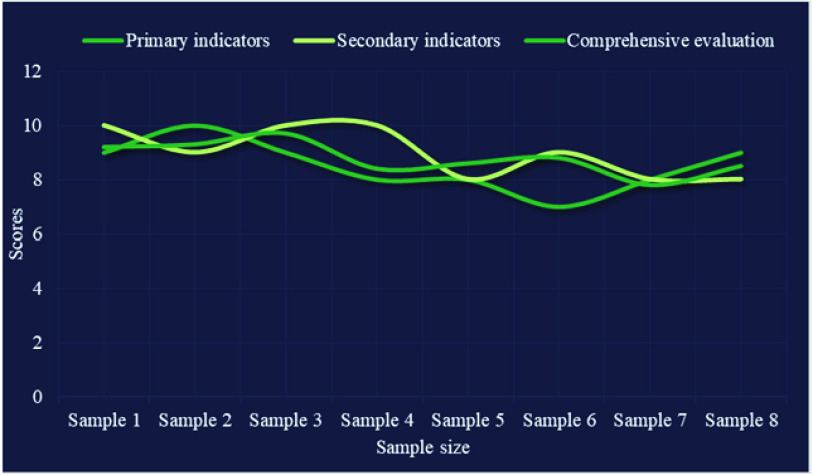
The first level index, the second level index and the comprehensive test evaluation results of the system.

Shown as Figure 4, according to the data collected from the test results of the evaluation system, 8 samples were randomly selected as the research objects as the training samples of this experiment. The closer the score is, the more accurate the description will be; otherwise, the greater the error will be. The results show that there is little difference between the scores of the first level index and the second level index and the comprehensive score. The validity of the test results of the system is verified, and the expected evaluation results are obtained.

### D. Comparison of Emergency Management Value Between a Provincial Government and Local Government

**FIGURE 5.**
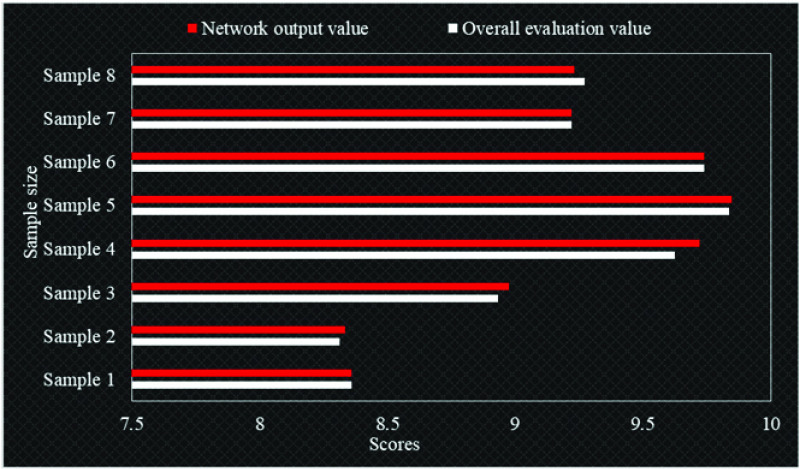
Comparison of evaluation value and actual value of emergency management system.

Shown as Figure 5, in order to verify the authenticity of the test results of the system, we call 8 test sample data and input them into the system. Through the deepening effect of BP neural network, the network evaluation value is calculated and compared with the overall evaluation value, so as to test the effectiveness of the intelligent evaluation result of the emergency system. Generally speaking, the results detected by the government emergency management intelligent evaluation system have little difference with the actual value, and the error between most network evaluation value and overall evaluation value is less than 0.05. This shows that the system evaluation effect after deepening BP neural network is true and reliable, which can truly reflect the emergency management level of government emergencies, and comprehensively evaluate the emergency management system through various indicators.

## V. Conclusion

The results of the novel coronavirus pneumonia prevention and treatment in 2019 have achieved good results, which shows that our country attaches importance to its management mechanism in the face of emergencies. However, there are also many problems in the emergency management system of low-level provinces and cities through the epidemic, such as lack of common sense, weak early warning, insufficient strength with the Internet of things, insufficient mobilization of personnel, weak social influence and low level of citizen participation. Therefore, it is imperative to improve the emergency management system of all levels. This paper starts from the emergency management system, combined with the basic situation, obtains the current situation of the emergency management system under the economic and social background, and through the comparison of the commonly used evaluation methods of the current emergency management system, it is proved that the BP neural network method has certain advantages in the establishment of the emergency management system. The paper also compares the scoring status of the staff before and after the application of the system in a province. The intelligent evaluation system of emergency management based on BP neural network is more effective, and the score is more prominent in the process of continuous improvement with time. In addition, the comprehensive evaluation value and network evaluation value of eight test samples are compared. It is found that the results detected by the emergency management intelligent evaluation system are similar to the actual value, and the error between most of the network evaluation values and the overall evaluation value is less than 0.05. It shows that the evaluation results of BP neural network deepening evaluation system are better, and the evaluation value is closer to the real value. Therefore, through the follow-up improvement, the performance and efficiency of the system can be improved, and the most reliable results can be obtained in evaluating the emergency management system of each place.
